# Indiscriminate Use of Alcoholic Stimulants in Disease

**Published:** 1867-08

**Authors:** Samuel Wilks

**Affiliations:** Physician to, and Lecturer on Medicine at the Hospital


### INDISCRININATE USE OF ALCOHOLIC STIMULANTS IN DISEASE. A Lecture Delivered at Guy's Hospital, London.

By SAMUEL WILKS, M.D., Physician to, and Lecturer on Medicine at the Hospital.

In drawing your attention to the case of bronchitis, and the good results which followed the discontinuance of stimulants after I had at first ordered them, let me impress upon you the importance of always seriously considering the advisability of alcoholic treatment before you have recourse to it. To my mind, the most important question in therapeutics at the present day is the value of alcohol in disease. Because this agent is made use of daily by a large part of the community as an article of diet, its administration in disease is treated too often with carelessness, and it thus forms, without a due consideration of its action, one of our commonest prescriptions. If it be said that its frequent use is an evidence of its potency, this is the more sufficient reason why its administration should be watched with the extremest care.

You know it is not decided in what manner alcohol exactly behaves in the animal economy; the proof is wanting that it is a food for the lungs, whereas its direct effect on the nervous system is evident. A want of knowledge of the precise changes which it undergoes in the system is, however, no argument against its use, since its advantages must be discovered from experience alone. If the taking a stimulant restores a flagging nervous force, and so adds fresh life to the various organs of the body, a benefit may have been received; and thus most of us are brought up in the habit of taking our glass of wine, and feel ourselves refreshed. Since the active agency in many of our stimulating drinks is alcohol, many persons have recourse to spirits in order the better to obtain its influence, and thus brandy may become with them a regular article of diet: the necessity for some stimulus being assumed, if malt liquor or wine be supposed to disagree, some brandy and water is drunk instead.

I am not going to enter upon the much-vexed question as to the necessity for the daily use of wine or spirit; but I will say that the usual test for the advantges of its use is one which I conceive to be in most cases utterly valueless. Alcohol, remember, although an excitant, is a sedative to the nervous system—is, in fact, an anæsthetic. A drunken man may be injured in such a way as to have all his teeth knocked out in a brawl, yet apparently not perceive the injury, and be utterly unconscious of the occurrence when he has returned to soberness. The argument, therefore, that a man feels better after his glass or two of grog would be equally applicable to the case of the Turk, who feels better for his opium. His feeling better simply means that he has got rid of his unpleasant sensations, whether these be moral or physical; he "drowns his trouble in the bowl." If a man engaged in the practice of his profession, mercantile business, or even pleasure, such as boating, cricketing, or shooting, intends to assert that he can pursue these respective objects with more success after he has taken a stimulant, then he may have an argument in favor of its use. If, however, it be admitted that during the active pursuits of the day a stimulation to the nervous system is injurious, but that after the fatigues are over the body must be recruited, and that a proportion of alcohol is beneficial, I have nothing to say against it, should experience speak in its favor. I repeat, however, that in the majority of instances where a man's reason for taking his wine or spirits is of no better kind than he feels better for it, the reason is utterly valueless; indeed, it may generally be assumed that whilst his feelings are benumbed his organization is being injured. The argument is no better in favor of the use of wine or other stimulant in disease—as, for example, that it must do good, since the patient craves for it. The question of the advantages attending the daily use of beer, wine, or spirits, although a difficult one to solve, is one which you cannot evade considering, since the health and welfare of families may depend upon your judicious decision. You may recommend wine with advantage to members of certain families having peculiar temperaments, while should you advise it for others you may unwittingly be sowing the seed of ruin of mind, body, and estate.

The subject of the different temperaments of your patients and their mode of life is one which is well worth your study; but the matter I wish now to strongly enforce upon you is that you are as thoroughly to consider the propriety of the administration of alcohol as you would any drug in the Pharmacœia. Endeavor, if you can, to erase from your minds that it is a proven fact that alcohol is a tonic or a necessary part of every one's beverage. This is assumed by a large mass of people; and the meaning of the question which your patient puts to you when he says, "What shall I drink?" is not, "Shall I take a stimulant or leave it alone?" but "Shall I drink beer, wine, or spirits?" He often confesses that he is in a great difficulty; he finds none of them agree with him; but that he must take "something" appears as necessary as eating his daily bread; the alternative never having formed part of his calculation. I say it is assumed that a strength-giving property lies in these drinks—that just in proportion to a man's feeling of weakness, so will he require one of them: in ordinary health he may not only want his beer; but if ill, his wine; and if very ill, his spirits. Now this popular opinion is shared in, I am sorry to say, by many in the profession: if the patient is weak he wants "support," this term carrying too frequently with it the necessary idea of wine or spirits. I should be sorry to say that the doctor panders to the public taste, since he is too often already in accord with it; but the consequence of such agreement between patient and medical man resolves itself into this, that an extra siimulant is prescribed. You might ask to what complaints do I refer when I speak of this too common advice; but I need only repeat the word "patient," for it matters little what is the nature of the disease, since the reasons for the treatment are applicable to all complaints, and are founded on this simple proposition: all persons who are ill are weak; they have lost strength; they require it to be restored; alcohol is a supporter and a tonic, therefore alcohol is a remedy for all diseases. This is no parody, for I have heard the argument set forth in some such words; and practically it is adopted by many, for I constantly hear medical men say they give brandy to all their patients, for they always find them "low." Brandy, indeed, becomes with some as much a universal remedy as revalenta, chlorodyne, Morison's pills, or any other quack medicine. Moreover, it is a medicine of which the patients approve, assuming as they also do its supporting and strength-giving powers. You therefore cannot do better, if you fear no compunctions in converting your profession into a mere trade, to say to all your patients, after feeling their pulse, that they are very low—that you are sure they do not take enough; and order them several glasses of wine daily. Should they be exceedingly ill with some desperate organic complaint, then you must turn your remarks to the friends, and speak of the necessity of supporting the patient by giving him as much brandy as can be poured down his throat. By this method you are sure to give "satisfaction:" for should the patient die without such treatment, you may have the credit of letting him slip "through fingers;" whilst, if he die with it, you have done your best. If you kill a dozen patients with brandy, you need have no fear—"you have done your best." This, I say, would be a very comfortable and lucrative mode of practice.

It may very fairly be asked, if alcohol be so potent a remedy that it can supersede all drugs in so many several cases of disease, is not this a reason why brandy-treatment should be adopted with some consideration? The want of caution in its use is owing, no doubt, to its entering so frequently into the daily diet; and thus alcohol is not reckoned amongst the same class of agents as that of medicines. On the bed-cards in this hospital there is one column for the medicines, and another for the diet. Before filling up the one, we discuss the benefits of giving our patients a few drops of henbane or ether; and in the other column we often write down any number of ounces of brandy with very little thought of its effect. If alcohol were transferred to the medicine side of the card, we should be more likely to discuss its value in any given case in the same manner as we do the various drugs in the Pharmacopœia.

It would require a whole course of lectures to dwell upon the beneficial or baneful effects of alcohol in all forms of disease; and, therefore, I will state, as a result of my own experience, that, like other drngs, it may be beneficial, useless, or harmful. I may remind you of what you yourselves have witnessed—that fevers will do well without this remedy. So wedded, however, are some to the idea of the absolute necessity of stimulants, that they have expressed almost incredulity when they have heard it stated that fevers will terminate favorably without them. Of course stimulants are often needed; but young persons with typhus and typhoid do far better, I believe, without them. That they make good recoveries on simple milk diet is a fact which my hospital cases prove, and which no arguments can gainsay; and, on the other hand, I have seen a marked improvement take place in some cases where a stimulus had been left off, It is also a fact that in bronchitis I have repeatedly seen improvement after stimulants have been omitted; and, as regards heart-disease, I am convinced that the amount of mischief done by stimulants is immense. In the case of fevers and bronchitis, the weak pulse is often but an indication of extreme capillary congestion, and a stimulus to the heart only aggravates the evil; and in the case of a diseased and weak heart, where repose is indicated, a constant stimulation by alcohol adds immensely to its trouble.

It causes me daily surprise to observe how the effects of stimulation are overlooked. Often have I been called to see a patient apparently dying, sometimes of a nervous disorder, at another time of a liver complaint, and at another of heart-disease. He is lying in bed, where he (or she) has been for some time, and kept alive (as it is said) by brandy; the breath is abominably fetid; the heart's action is so rapid that it is impossible to say whether the organ is diseased or not; the patient refuses food, or if this be taken, it is rejected, and so he is plied with brandy to keep him alive; the body is, in fact, saturated with spirits, or its elements. My first remark on seeing such a case is, that a man cannot live on alcohol; he must take some food or he will die. The correctness of such common-sense remarks is admitted, but qualified with the statement that no solids can be taken, and that if stimulants be omitted it is feared the patient will sink. It is assumed that the constant administration of brandy is necessary for the temporary maintenance of life, and the idea never seems to have been conceived that the stimulation of the heart causes the weak, fluttering pulse, and the stimulation of the stomach a subacute gastritis. Do you ask me what method I adopt? The simplest possible. I withdraw every drop of the stimulant, and in a few hours the irritated stomach is partly restored to its normal condition, the nervous excitement abates, the patient takes a little food, and begins to mend. Do you ask, again, whether I do not fear any frightful result from the sudden withdrawal of the stimulus? I say, not the least; I have no fear of the consequences. Not of delirium tremens? Not in the least. This is a disease not induced by the withdrawal of stimulants, but, on the contrary, is produced by a recent debauch. For the production of delirium tremens the patient must have been such a habitual tippler as to have weakened his brain, and must then have had an overdose of the stimulant to set up the disease. There are no facts to show that the withdrawal of the accustomed drink is attended with any evil results, although I know that an imaginary fear of this kind leads to an erroneous and vicious method of treatment—the plying the patient with a stimulant during the violence of the attack, the effect of which is to prevent or prolong the cure. Rest and repose, with the avoidance of stimulation, is the treatment which the patient requires. So success of digitalis may be mentioned in corroboration of this view. I repeat that there are no facts to show that delirium tremens is produced by the withdrawal of stimulants, whilst it is a fact, as I could illustrate by many cases, that nothing but good results from its absolute discontinuance in the desperate cases to which I have alluded.

That many cases of disease of various kinds would do far better without stimulants I am perfectly confident. But lately I have seen the case of a gentleman, about sixty years of age, who passed through a most severe attack of pneumonia without the use of stimulants. He had been a tolerably free liver, and would not have been called a good subject; but having before me the case of another gentleman of the same age, who had just died of pneumonia, and who had taken a large quantity of brandy, I readily acquiesced in the patient's own view, that none should be given. It is very remarkable what extremes we have reached, and on how slight a scientific bases is founded the treatment of pneumonia. Not many years ago the antiphlogistic method was adopted, including bleeding, antimony, calomel, etc.; then came the "let alone" method; and now we have the brandy treatment. What the need of this can be, with Professor Hughes Bennett's statistics before us, I do not comprehend. My own opinion is (but of course this is only an opinion) that in any given number of cases a larger majority would recover under the old antiphlogistic treatment than by the more modern remedy by brandy. As regards heart-disease, the utmost discrimination is required in the use of stimulants. There are cases where an undoubted benefit is produced by them; but there are others, and these I have seen repeatedly, where alcohol has induced palpitation, fluttering, great distress, and constant sleepless nights, but where, on the other hand, the withdrawal of the spirit, and the substitution of a dose of digitalis or henbane, has been of the most essential service. The administration of a stimulus, in the attempt to overcome disease, in lieu of good and well-tried remedies, evinces the very worst form of medical scepticism with which I am acquainted.

It is not only in these severe cases of disease, but in lesser troubles, that your recommendation of stimulants may do incalculable mischief. You visit, for example, an ailing lady, and she details to you a number of troubles of a nervous and dyspeptic character. She is sitting in-doors all day, taking no exercise, living well, and consequently drifting into a weak and flabby condition. You place your hand on her pulse, and, finding it feeble, condole with her on her state of health, assure her that she does not live well enough, and order her a few extra glasses of wine or a little brandy.* You find that she grows no better for the advice; but perhaps you never reflect that you have been adding fuel to the fire. Knowing not what to do in the way of treatment, you order her out of town, and she immediately begins to improve. She goes to Brighton, rides on horseback, or walks miles a day on the Parade, regains her appetite, craves less for stimulants, and her health is restored. If, on the contrary, you fail to remove her from her home, she goes on from bad to worse; she takes to her bed, eats less food, drinks more wine and brandy, until, having become one mass of fatty degeneration, life can hold no longer, and death ends the scene. This lady has been killed with kindness. This is no imaginary case; my mind's eye carrying me to the bedside of more than one such instance. Do not then assume that alcohol is equivalent to a tonic, and that it must be necessarily administered because your patient is weak. It may be that very weakness is due to the long-continued pernicious effects of this same stimulant; indeed, as you have often heard me say in the out-patient room, if a man comes into our presence with a tottering gait, bloated face, and his nervous energy all gone, you may be quite sure that he has been taking "strengthening" things all his life.

I will say no more on the subject, as I do not wish to speak demnatory of alcohol as a remedy, since it is one of the most powerful agents we possess to rouse the dormant nervous power.

* The word "little," it must be remembered, has long ceased to maintain its original signification in reference to eating, drinking, and physicking. It would be extremely vulgar were we to be asked at our dinner-tables to take otherwise than a "little" more; and the doctor would not be forgiven by his patient, were he, in detailing the ingredients of his prescription, to state that he had administered the regular dose, but that he had given only a "little" of this or that. When, therefore, a patient is ordered a "little" brandy, the adjective in no way qualifies the amount.

Moreover, I do not wish to speak too dogmatically of its ill-effects, fully aware that there are many holding very distinguished positions in the profession whose opinions are not in accordance with those I have expressed. Were it not for this reason, I should have used still stronger language than I have done; for even firm convictions must be restrained, when we know what an amount of contrary opinion can be arrayed against us. It is, nevertheless, the duty of every one to express his own conviction when that is based on experience, and thus I shall ever feel bound to withstand the indiscriminate use of stimulants in disease.

Whatever may be thought of the remarks just made, there is one thing which I must insist upon—that is, when treating any malady, and the administration of alcohol is suggested to your mind, that you give the same grave consideration to its recommendation as you would to any other potent drug in the Pharmacopœia; not to sit down and give all your serious thoughts to the question of whether a grain of this or a grain of that drug should be ordered, perhaps twenty or thirty drops of ether, and then at haphazard order any loose number of ounces of brandy. You observe that I say nothing against the potency of alcohol in several states of disease; but I do speak strongly against its indiscriminate use, without due consideration of its need or of its results. My arguments would equally apply did I find that opium or any other drug were indiscriminately used as a universal medicine. I should protest against the practice, whilst still possessing great faith in the virtue of the drug. If I can influence you to place alcohol in your list of drugs, so that you may administer it with the same caution as you do the several articles in the Pharmacopœia, then the object of these remarks will be fully answered.—*London Lancet.*

